# Functional aging in health and heart failure: the COmPLETE Study

**DOI:** 10.1186/s12872-019-1164-6

**Published:** 2019-07-30

**Authors:** Jonathan Wagner, Raphael Knaier, Denis Infanger, Konstantin Arbeev, Matthias Briel, Thomas Dieterle, Henner Hanssen, Oliver Faude, Ralf Roth, Timo Hinrichs, Arno Schmidt-Trucksäss

**Affiliations:** 1 Department of Sport, Exercise and Health, University of Basel, Birsstrasse 320 B, 4052 Basel, Switzerland; 2 Biodemography of Aging Research Unit (BARU), Social Science Research Institute, Duke University, Durham, NC 27705 USA; 3 Department of Clinical Research, Basel Institute for Clinical Epidemiology and Biostatistics, University Hospital Basel, Spitalstrasse 12, 4056 Basel, Switzerland; 4 Department of Health Research Methods, Evidence, and Impact, McMaster University, 1280 Main Street, Hamilton, ONL8S4K1 Canada; 5 University Clinic of Medicine, Cantonal Hospital Baselland, University of Basel, Rheinstrasse 26, 4410 Liestal, Switzerland

**Keywords:** Aging, Fitness, Exercise, Vascular function, Heart failure

## Abstract

**Background:**

Cardiovascular (CV) diseases including heart failure are the leading causes of morbidity, with age being the primary risk factor. The combination of age-related organic functional impairment and reduced physical fitness can drastically impact an individual’s healthspan. One’s lifespan can potentially be prolonged by the preservation or improvement of physical fitness. However, it remains unclear as to which biomarkers are most suitable for distinguishing between healthy aging and the impaired organ function associated with heart failure. Therefore, a comprehensive assessment of the components of physical fitness and CV function will be performed to identify the most important factors contributing to aging in relation to both health and disease.

**Methods:**

This cross-sectional investigation will consist of two parts: COmPLETE-Health (C-Health) and COmPLETE-Heart (C-Heart). C-Health will examine the aging trajectories of physical fitness components and CV properties in a healthy population sample aged between 20 and 100 years (*n* = 490). Separately, C-Heart will assess the same markers in patients at different stages of chronic heart failure (*n* = 80). The primary outcome to determine the difference between C-Health and C-Heart will be cardiorespiratory fitness as measured by cardiopulmonary exercise testing on a bicycle ergometer. Secondary outcomes will include walking speed, balance, isometric strength, peak power, and handgrip strength. Physical activity as a behavioural component will be assessed objectively via accelerometry. Further, CV assessments will include pulse wave velocity; retinal, arterial, and venous diameters; brachial and retinal arterial endothelial function; carotid intima-media thickness; and systolic and diastolic function. The health distances for C-Health and C-Heart will be calculated using the methodology based on statistical (Mahalanobis) distance applied to measurements of quantitative biomarkers.

**Discussion:**

This research seeks to identify physical fitness and CV biomarkers that best resemble underlying CV risk with age. Further, it will examine which physical fitness markers are impaired most in heart failure. The presented integrative approach could define new recommendations for diagnostic guidance in aging. Ultimately, this study is expected to offer a better understanding of which functional characteristics should be specifically targeted in primary and secondary prevention to achieve an optimal healthspan.

## Background

The population of industrialized countries is aging. By the year 2050, at least one-quarter of the population in developed countries will be older than 65 years of age [1]. Similarly, life expectancy is projected to increase in industrialized countries with probability rates of at least 65% for women and 85% for men, respectively [2]. A more significant part of the projected gains in life expectancy at birth will be due to enhanced longevity above the ages of 50 and 65 years [2]. In recent decades, aging and associated chronic diseases have become an increasing concern for our society and the health care system [3]. The evolutionary ‘unforeseen’ gain in lifespan would not be a catastrophe if it were a gain of healthy years of life. However, since the leading causes of early mortality, such as death around birth and death from infections, can largely be prevented by modern medicine, the presence of chronic noncommunicable diseases at middle age to advanced age progressively gain in importance. Nonetheless, cardiovascular (CV) disease, which includes heart failure, is the leading cause of morbidity, and chronological aging is its primary risk factor [4, 5]. The prevalence of chronic heart failure (CHF) in the developed world has increased significantly in the last three decades [5]. CHF is associated with high morbidity and mortality, making this chronic condition a significant health care concern [5].

**Fig. 1:**
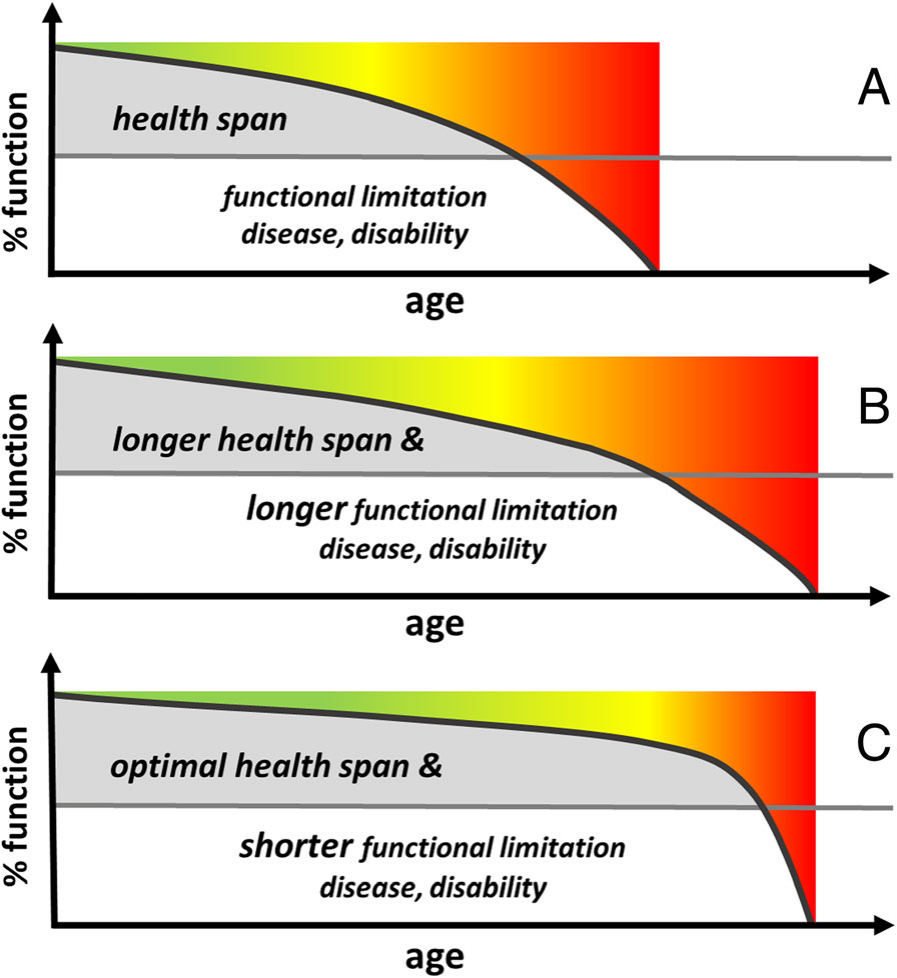
Healthspan. Healthspan is the period of life without functional limitations, chronic diseases. and disability but with a beginning loss of functional capacity symbolized by a change from green to orange colour (**a**). This period may be prolonged by curative medicine (**b**), but longer disability may also occur. Optimal healthspan can potentially be achieved by preserving or improving the functional abilities of the organism and therefore lead to a shorter period of functional limitations, chronic diseases, and disability at the end of life (**c**). Good physical fitness is thought to contribute essentially to optimal healthspan. Adapted from Seals et al. [7]

A concept to counteract the traditional way of thinking of age-associated chronic diseases was introduced as early as 1980 [6]. The goal of this concept was to increase the years free of disease or to shorten the disease phase during the final part of one’s life. The compression of morbidity is to be attained by optimal prevention [6] and presumes that illness is absent up until a certain point in life and then is present for a brief period thereafter until the end of one’s life. Since this concept neither adequately takes into account the aging of the organism nor considers the functional limitations that may present before the onset of a disease, the term ‘healthspan’ was recently introduced [7–9]. One’s healthspan is defined as a period of relatively healthy aging followed by a period of age-related diseases and disabilities [7–9] (Fig. 1 a). During the period of relatively healthy aging (orange to red area in Fig. 1 b), the functions of various organ systems are already somewhat restricted, including the CV system. Vice versa, the diminishing function of the organ systems due to age increases the risk of chronic diseases.

Curative medicine prolongs a lifespan but may also be associated with a longer time of disability (Fig. 1 b). An optimal healthspan may be achieved through the combination of curative medicine as well as an improvement of one’s physical fitness components (Fig. 1 c).

Physical fitness is defined as a set of attributes (e.g., endurance capacity, muscle strength, neuromuscular coordination) that people have or attempt to achieve in order to carry out daily tasks without undue fatigue [10, 11]. Reduced physical fitness is accompanied by a reduction in the use of the organ systems involved in physical activity and appears to be equally important as the aging of the organs themselves in the concept of healthspan. The deterioration of the main components of physical fitness (i.e., endurance capacity, muscle strength, and neuromuscular coordination) due to inactivity or insufficient physical activity is associated with a lower capacity of the CV system, skeletal muscles, and the neuromuscular system [12, 13]. The final state of this unfavourable process is generally regarded as a frailty or disability status [14].

The age-associated functional limitations of the organ systems and the lower level of physical fitness due to reduced physical stimuli cannot clearly be separated from one another but rather interact with one another in the aging process [15]. In addition, acute or chronic illnesses affect physical fitness and can accelerate the effects of reduced use and thus cause the organism to reach the threshold of frailty more quickly [7, 16]. On the other hand, an improvement of the physical fitness can alleviate the severity of occurring diseases. The overarching idea related to these two processes (decline of physical fitness and age-associated functional limitations of the organ systems) is now to use their interaction to counteract the development of chronic diseases by maintaining or improving physical fitness. This is all the more likely because physical activity and training on numerous paths can influence the development of the disease and its course, which has been proven many times [17, 18].

The combination of age-related organic functional impairment and reduced physical fitness can debilitate the healthspan of an individual. Separately, the healthspan can potentially be prolonged by the preservation or improvement of physical fitness with advancing age.

The rationale for a combined assessment of the aforementioned components of physical fitness arise from their individual and separate predictive values for all-cause mortality and CV mortality [19–22].

The isolated assessment of physical function would be, however, worthless to perform in the described cross-sectional study without robust surrogate health markers. As it is impossible to test all physiological organ functions against the physical function parameters, this study instead focuses on vascular health and CV disease surrogate markers to characterize organ function. CV biomarkers have been shown to be excellent markers for overall physiological status during aging. Arterial dysfunction increases the risk of several common chronic disorders with advanced age such as coronary disease, kidneys disease, stroke, cognitive impairment, Alzheimer’s disease, motor disorder, and heart failure [23]. Impaired physical fitness is a ‘gateway’ to early vascular aging, vascular dysfunction, and an increased risk for CV disease [19].

**Fig. 2:**
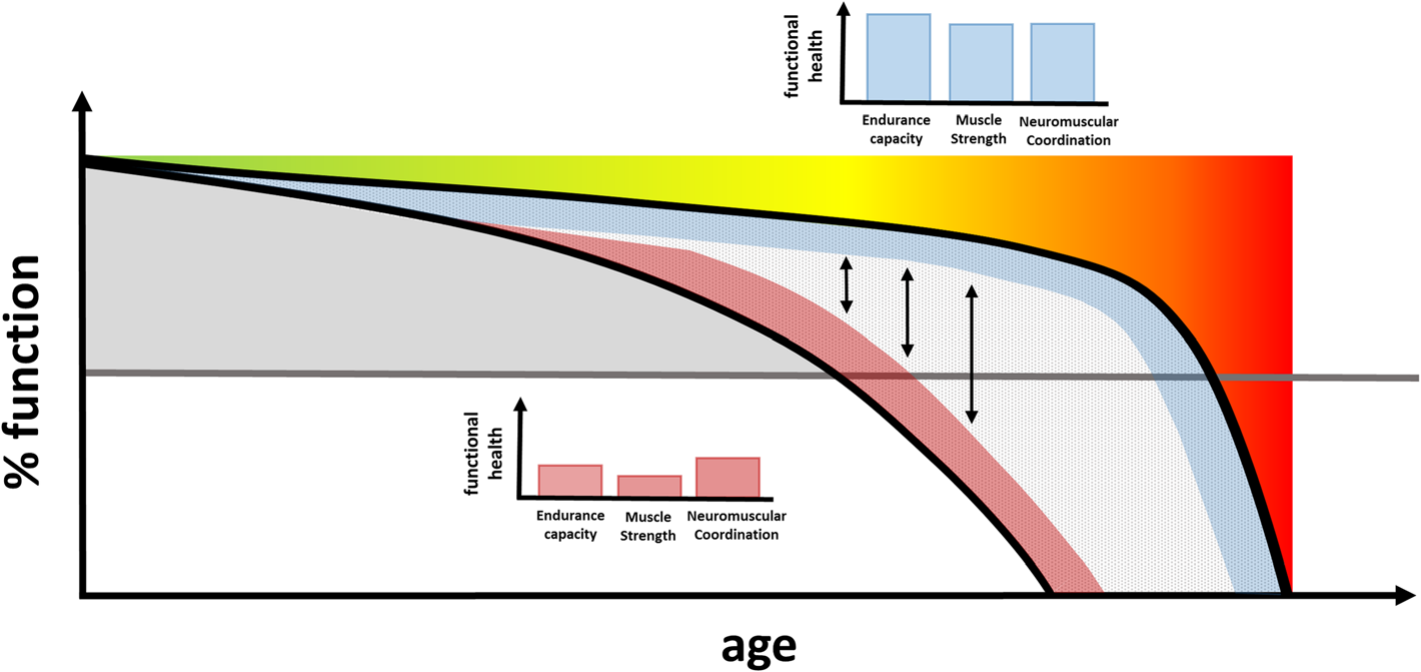
Health distance. Graphical illustration of the health distance. Healthy individuals are potentially located at the upper limit of the healthspan (blue area). Individuals with heart failure are potentially located at the lower limit of the healthspan (red area) and reaching the line of functional limitation (grey line) earlier as compared with healthy individuals. Double arrows denote the health distance between healthy individuals and patients with chronic heart failure. The upper bar chart represents the better components of physical fitness of healthy individuals, while the lower bar chart represents the worse components of physical fitness of a patient with chronic heart failure. Modified from Seals et al. [7]

It is still unclear as to which CV and physical fitness biomarkers distinguish best between healthy functional and dysfunctional aging. Also, the potential variations with age of these markers in healthy people and in people with early stages of chronic disease such as heart failure are largely unknown (Fig. 2). A thorough assessment of vascular health, CV diseases surrogate markers, and fitness parameters is needed to understand the relationships and interplay between physiological function and physical fitness.

## Physical fitness components and aging in health and heart failure

### Endurance capacity

Several prospective studies on endurance capacity show a strong predictive value for overall and CV mortality. Kodama et al.’s meta-analysis [19] involving 102,980 participants and 6,910 outcomes for all-cause mortality revealed a substantial contribution of cardiorespiratory fitness that was independent of classical risk factors. As compared with participants with high fitness, those with low fitness had a 70% higher risk for all-cause mortality and a 56% higher risk for CV mortality. A recently published follow-up study of 22,878 participants with a baseline mean [standard deviation (SD)] age of 47.4 (10.3) years and a follow-up of 9.2 (SD: 4.1) years and 505 deaths added cardiorespiratory fitness to the Systematic Coronary Risk Evaluation (SCORE) risk model. The study revealed a substantial improvement of the combined risk model of SCORE. Cardiorespiratory fitness alone was also a better risk predictor than the SCORE value alone [20]. People with high SCORE values and low fitness (metabolic equivalents < 11) showed a relative hazard ratio for death of 35.6 versus the low SCORE value and high fitness group. However, those with high SCORE values but high fitness (metabolic equivalents ≥11) had only a hazard ratio of 8.5. A recent analysis of a Finnish population confirmed these results for incident fatal myocardial infarction and CHF. From 2,089 participants with a baseline age of 53.1 (SD: 4.9) years and a follow-up of 19.1 (SD: 8.4) years, the rate of fatal myocardial infarction (*n* = 522) and nonfatal heart failure increased with a reduction in cardiorespiratory fitness [24]. The Mayo Clinic’s patient database recently included cardiorespiratory fitness in a treadmill score in the risk prediction for CV mortality. Out of 58,020 participants with a mean age of 53 years (49% women), 6,456 patients (11%) died by the median follow-up point over 10 years. When cardiorespiratory fitness was considered in the risk prediction, traditional CV risk factors did not contribute incrementally to survival discrimination [25].

In heart failure, the most important and often used parameter for risk prediction is peak oxygen uptake (V̇O_2_peak). Different studies have shown an incremental value of V̇O_2_peak to other risk factors [21, 26, 27]. V̇O_2_peak and percent-predicted V̇O_2_peak added prognostic value when included in a multivariate Cox regression analysis with standard risk variables including New York Heart Association (NYHA) functional class and left ventricular ejection fraction (EF) (LVEF) [21]. This incremental value of V̇O_2_peak has also been shown for heart failure with a preserved EF (HFpEF) [28]. The relationship of the ventilation to carbon dioxide production slope (V̇E/V̇CO_2_ slope) is a second independent predictor for CHF hospital admission after consideration of recognized clinical variables such as age, NYHA functional class, EF, body mass index, creatinine, and B-type natriuretic peptide (BNP) [27]. The variables of cardiopulmonary exercise testing (CPET), exercise oscillatory ventilation (EOV), oxygen uptake efficiency slope (OUES), and the partial pressure of end-tidal CO_2_ (PETCO_2_) constitute additional variables that have all been prospectively validated [29]. As described in a recent review [29], each variable reflects in part a different pathophysiologic feature of heart failure with reduced EF (HFrEF) and therefore recommends the parallel assessments of these five CPET variables in those patients.

### Muscle strength

Muscle strength is determined by muscle mass and fibre composition as well as intra- and intermuscular coordination. Muscle power decreases more significantly in men and women from the age of 40 years onward, a phenomenon that can only partly be explained by the loss of muscle mass [30]. Rather, the change in fibre composition is decisive for the contraction velocity that determines muscle power. Muscle strength reaches its peak around the age of 30 years and remains almost constant until the age of 50 years and then decreases continuously between 2 and 5% per year depending on the individual’s age [31]. The loss of muscle mass and strength that occurs with advanced age is defined as sarcopenia. Traditionally, sarcopenia has been defined as a loss of appendicular muscle mass of less than two SDs below the mean muscle mass of a person aged 35 years old [32]. Other thresholds used for the definition include low grip strength or a low usual gait speed of less than 0.8 to 1 m/s [14, 33, 34]. Sarcopenia syndrome is characterized by a progressive and generalized loss of skeletal muscle mass and strength and associated with an increased risk of adverse outcomes such as physical disability, poor quality of life, and death [35]. Grip as well as leg strength have been shown to be inversely associated with mortality [36]. Muscle strength seems to be a better predictor than muscle mass [22]. Recent research shows that the prediction of mortality and hospitalization based on different upper and lower limb measurements is superior to the measurement at one location [37], whereby the predictive power depends on the disease [38].

Skeletal muscle mass is reduced in heart failure with reduced EF by approximately 20% and in heart failure with preserved EF by approximately 10%, respectively [35, 39]. According to the NYHA classification scheme, significant differences in muscle strength have also been demonstrated in patients with heart failure, with severity levels differing. For example, the strength of the leg extensors and flexors as well as the hand grip strength are significantly decreased in patients with NYHA functional class III versus those with NYHA functional class I [40].

Furthermore, a significantly lower strength of the *M. quadriceps* femoris and of the hand grip is found in heart failure patients both with and without muscle wasting [39, 41]. Even in the mild stages of heart failure, leg strength is already limited [41]. In addition to the reduction of muscle mass, a reduction of type I fibres can be observed in the skeletal muscles [42]. This applies to patients with either HFrEF or HFpEF as compared with healthy controls [43]. These changes in the peripheral musculature seem to be mainly responsible for the reduction of endurance capacity. An improvement in V̇O_2_peak is found to be 27% by the muscle oxygen diffusion capacity and only about 7% by the cardiac output [44].

In summary, muscle wasting and sarcopenia in people with heart failure may be noninvasively detected through impaired physical fitness such as reduced muscle strength.

### Neuromuscular coordination

Neuromuscular coordination is typically assessed by balance testing and gait analysis [45]. In older people, gait speed is a strong independent predictor of mortality [46]; the United Kingdom Biobank study confirmed that habituary walking speed is one of the strongest predictors of mortality in both sexes [47]. However, the change in walking speed in older people is preceded by other changes in gait characteristics such as gait variability, cadence, stride length, and stride width that begin to occur in middle age [48, 49]. The relationship between muscle strength and gait characteristics were investigated in the Baltimore Longitudinal Study of Aging in middle-aged (32–57 years), old-age (58–78 years), and ‘oldest’-age (79–93 years) participants. A reduction in walking speed between middle age and old age was found to be a result of a reduction in muscle strength and neuromuscular coordination [13]. Beyond the age of 60 years, slow walking speed is an independent and strong predictor of poor health status. Further, poor balance and mobility are significant predictors of mortality in predisabled women aged 75 years and older [50].

In heart failure patients, gait speed is an independent predictor of mortality [51]. In a recent study, gait speed was associated with a lower risk for all-cause mortality independent of age; EF of less than 20%; and other parameters such as systolic blood pressure, anaemia, and the absence of beta-blocker therapy [52].

## Cardiovascular phenotype in health and heart failure

### Vascular biomarkers

The assessment of vascular biomarkers is essential in order to research the potential impact and link between physical fitness and physical activity on vascular function and/or vascular impairment. In addition to a broad spectrum of measures of physical functioning, the assessment of vascular biomarkers and quantification of the burden of atherosclerosis in asymptomatic, apparently healthy individuals and heart failure is also of considerable importance.

In 60% of asymptomatic individuals aged 68.9 ± 6.0 years, subclinical atherosclerosis was detectable in two vascular beds (carotid and coronary arteries) [53]. Additionally, the MONICA–Augsburg study found at least one plaque in the carotid or femoral artery in 51.8% of men and 36.3% of women assumed to be healthy [54]. Since few studies have shown an inverse association of prevalent subclinical vascular disease and components of physical fitness [55–58], it is crucial to assess the atherosclerotic burden in parallel.

### Arterial stiffness

Arterial stiffness increases with age and is measured as pulse wave velocity (PWV). The increase in arterial stiffness accelerates in particular beyond the age of 60 years [59]. The increase in PWV causes an increase in central arterial blood pressure and is thought to cause left ventricular hypertrophy and impaired left ventricular diastolic function [60]. Increased PWV was identified as an independent predictor of CV and all-cause mortality in a prior meta-analysis of individual data [61]. Vigorous physical activity and higher cardiorespiratory fitness are inversely associated with age-related arterial stiffening [55]. Improvements in cardiorespiratory fitness may, therefore, be a useful measure for preventing age-related increases in arterial stiffness [62].

In HFpEF, brachial–ankle PWV (baPWV) demonstrated a J-shaped association with CV events in a Japanese study. The authors suggested a low baPWV in HFpEF patients reflected aggravated cardiac diastolic dysfunction, while a high baPWV indicated large-artery stiffening [63]. A recent prospective study performed among heart failure patients from the Health, Aging, and Body Composition study did not show any independent predictive value of central PWV for risk prediction [64]. However, it is still possible that other parameters of central haemodynamics—for example, central pulse pressure—may have a predictive value. Another study showed an improvement in physical function in heart failure patients following vasodilator therapy and improvements in central haemodynamics [65]. PWV is predictive of left ventricular hypertrophy and CV events in hypertensive patients regardless of hypertension status (controlled or uncontrolled blood pressure) [66]. Therefore, PWV represents a promising marker with which to characterize and distinguish healthy aging and early stages of heart failure.

### Brachial artery flow-mediated dilatation

Impaired flow-mediated dilation (FMD) reflects endothelial dysfunction as an early marker of atherosclerotic arterial damage [59]. In addition to being sensitive to changes in lifestyle [67], FMD is associated with traditional risk factors, CV diseases, and heart failure and predicts both CV events and all-cause mortality [59]. Impaired FMD is associated with reduced exercise capacity, aging [62, 68], and heart failure. Impaired FMD in heart failure improves with drug therapy [69].

### Retinal vessels

Retinal vessels are regulators of the local cerebrovascular blood flow, are valid and robust microvascular surrogate biomarkers of CV risk and mortality, and can be analysed very effectively [70, 71]. Large cohort studies have previously shown that narrower retinal arterioles, wider retinal venules, and a resulting lower arteriolar-to-venular diameter ratio (AVR) are associated with an increased risk and severity of hypertension [72, 73], risk of stroke [71, 74] and CV morbidity and mortality in older participants [75]. In older adults, obesity is associated with retinal venular widening, and a lower AVR can be explained by the association of low-grade inflammation with obesity [76, 77].

To date, very little is known about the association of physical fitness with microvascular health. The only available exercise intervention study showed that higher physical fitness levels are associated with higher retinal AVR and that exercise-induced arteriolar dilatation as well as venular constriction lead to a significantly improved AVR in middle-aged lean and obese individuals [78]. In particular, the obese group seemed to benefit the most from the exercise training, with significantly dilated retinal arteries observed after a 10-week exercise program [78]. Dynamic retinal vessel analysis is a new innovative diagnostic method for the assessment of cerebrovascular endothelial function. It has recently been shown that retinal endothelial function, assessed as retinal vessel dilatation in response to flicker light, is impaired in prediabetic and diabetic patients as well as individuals with CV risk and CHF [79, 80].

To conclude, macro- and microvascular arterial properties are predictive of CV events and may help to discriminate between healthy individuals and patients with heart failure. Among motor functional components, endurance capacity specifically is inversely associated with vascular properties.

## Methods/design

### Objectives

This project seeks to do the following:

- Specific Aim 1

Determine the trajectories of physical fitness components of healthy aging by the measurement of endurance capacity, muscular strength, and neuromuscular coordination in a healthy population sample aged between 20 and 100 years.

- Specific Aim 2

Determine the health distances of physical fitness components (i.e., endurance capacity, muscular strength, and neuromuscular coordination) for patients with heart failure and age-matched healthy men and women.

### Study design

The COmPLETE study is a cross-sectional study and consists of two parts, COmPLETE-Health (C-Health) and COmPLETE-Heart (C-Heart).

### Hypotheses

Hypothesis 1 (C-Health): There is an apparent effect of age on all physical fitness components (endurance capacity, muscular strength, and neuromuscular coordination) in persons aged 20 to 100 years old.

Hypothesis 2 (C-Heart and C-Health): The health distances of components of physical fitness (endurance capacity, muscular strength, and neuromuscular coordination) for patients with heart failure and healthy individuals are not equal (Fig. 2).

### Recruitment

In C-Health, recruitment will be performed until a total number of 490 participants with a valid cardiopulmonary exercise test (CPET) as our primary outcome are included. All participants will be recruited in the area of Basel, including 35 males and 35 females per age category (i.e., 20–29, 30–39, 40–49, 50–59, 60–69, 70–79, and 80+ years of age). The recruitment will be based on unaddressed letters sent to randomly chosen districts of the 11 neighbourhoods of the city of Basel and 15 municipalities of the district of Arlesheim. Arlesheim is one of the five districts of the canton of Basel-Country. These districts represent both rural and urban environmental conditions. Potential participants in the age category of 80+ years will likely be underrepresented and only make up approximately 6% of the population living in this area only. Therefore, the recruitment will be widened for this group. After finishing the recruitment of all other age categories, recruitment letters will be sent to specifically targeted neighbourhoods with a higher percentage of older inhabitants but still involving rural and urban districts. Further measures for recruiting participants aged 80+ years might be discussed if necessary. A telephone questionnaire screening of potential participants before making appointments will be conducted. Final eligibility will be confirmed onsite on the day of examination.

C-Heart will include 80 heart failure patients characterized according to criteria named below. C-Heart participants will be recruited using various recruiting strategies such as recruiting letters, flyers; cooperation with a local cardiology unit of a hospital; and cooperation with internists and cardiologist in the area of Basel, Switzerland. The cardiologists and internists will provide potential participants with an information sheet about the goal and the conduct of the present study and identify interested patients for eligibility based on the inclusion and exclusion criteria. Potential participants through other recruitment channels will be reviewed for eligibility per telephone interview. For all potential C-Heart participants, the final eligibility will be confirmed onsite on the day of examination by a physician.

### Inclusion criteria

C-Health:

- Healthy men and women aged 20–100 years
  - ◦ Body mass index < 30 kg/m^2^
  - ◦ Nonsmoker

C-Heart:

- Stable CHF (treated patient with symptoms and signs that have remained generally unchanged for at least one month) characterized according to the European Society of Cardiology guidelines for the diagnosis and treatment of acute and chronic heart failure [81], as follows:
  1. HFrEF (LVEF < 40%)
  2. HFmrHF (LVEF 40–49%) and NT-proBNP > 125 pg/mL and relevant structural heart disease or diastolic dysfunction
  3. HFpEF (LVEF ≥50%) and NT-proBNP > 125 pg/mL and relevant structural heart disease or diastolic dysfunction

### Exclusion criteria

C-Health:

- Age younger than 20 years; manifest exercise limiting chronic disease (e.g., myocardial infarction; stroke; heart failure; lower-extremity artery disease; cancer with general symptoms; diabetes; clinically apparent renal failure; severe liver disease; chronic bronchitis GOLD stages II to IV; osteoporosis), women with known pregnancy or breastfeeding; drug or alcohol abuse; hypertonic blood pressure of more than 160/100 mmHg; compromising orthopaedic problems; Alzheimer’s disease or any other form of dementia; inability to follow the procedures of the study (e.g., due to language problems, psychological disorders, dementia of the participant); diseases regarded as an absolute contraindication for maximal exertion; and current or past smoking status.

C-Heart:

- Age younger than 20 years; women with known pregnancy or breastfeeding; drug or alcohol abuse; inability to follow the study procedures (e.g., due to language problems, psychological disorders, etc.); unstable angina pectoris; uncontrolled brady- or tachyarrythmia; permanent atrial fibrillation; severe uncorrected valvular disease; acute myocardial infarction or coronary syndrome; transient ischemic attack or stroke occurring less than three months prior; clinically significant concomitant disease states (e.g. uncontrolled hypertonic blood pressure); clinical evidence of current malignancy with exception of basal cell or squamous cell carcinoma of the skin and/or cervical intraepithelial neoplasia; currently receiving systemic chemotherapy and/or radiotherapy; significant musculoskeletal disease other than that associated with heart failure limiting exercise tolerance; active infection; immunosuppressive medical therapy; life-expectancy of less than six months; and prevalence of a disease regarded as an absolute contraindication for maximal exertion.

### Setting

The study will be carried out at the Department of Sport, Exercise, and Health at the University of Basel, Switzerland. This study is funded by the Swiss National Science Foundation (grant no. 182815) and was approved by the Ethics Committee of Northwestern and Central Switzerland (EKNZ 2017–01451).

### Study procedures and ethical considerations

The research project will be carried out in accordance to the research plan and with principles enunciated in the current version of the Declaration of Helsinki and the guidelines of Good Clinical Practice (World Medical Association, 2013).

The Ethics Committee of Northwestern and Central Switzerland and regulatory authorities will receive annual safety and interim reports and will be informed about study stop/end in agreement with local requirements. This study protocol was designed according to the Strengthening the Reporting of Observational Studies in Epidemiology guidelines. All measurements and procedures applied in this study are noninvasive.

All participants will be briefed verbally and will receive information approved by the local ethics committee giving details on the study procedures. All participants will have to sign a consent form and will be informed about their right to withdraw from the study without any consequences. Retinal vessel analysis will include mydriasis of one eye. Using a mydriaticum (tropicamide 0.5%), the pupils will be dilated to enable retinal vessel analysis. The eye drops can cause temporary discomfort, often a burning sensation for one to two minutes. Flicker light exposure can potentially cause slight headaches.

## Methods and course of measurements

The measurements will be carried out within approximately 3.5 to 4 h for visit 1 and 60 min for visit 2. The assessment of physical activity will be monitored for 14 days by a wrist-worn accelerometer after visit 1. Retinal vascular assessment will be carried out on a different day (visit 2) that takes 60 min and which is not more than one months apart from visit 1. The precise sequence is shown in Table 1. Standardized procedures will be used to perform all measurements, and the assessment staff will use standardized instructions for all measurements to ensure equal testing conditions for all participants.

**Table 1:** Outcomes assessed in the COmPLETE Study (C-Health & C-Heart)

	Outcome measure	Data Collection Instrument
Before Visit 1		
Telephone interview	General health and chronic disease, part 1^a^	21 items
	Smoking status ^a^	3 items
	Physical activity readiness ^a^	7 items, Physical Activity Readiness Questionnaire (PAR-Q)
Visit 1		
Questionnaires	Chronotype (1)	14 items, Munich Chronotype Questionnaire (MCTQ) [82]
	Quality of life (2)	8 items, Health related Quality of Life, short form (SF-8) [83]
	Socio-economic status (3)	1 item
	Subjective physical activity (4)	10 items, European Health Interview Survey-Physical Activity Questionnaire (EHIS-PAQ) [84]
		6 items, Global Physical Activity (GPAQ) [85]
	Residential area (5)	3 items
	Use of transportation (6)	3 items
	Life-space (7)	9 items, modified UAB Study of Aging Life-Space Assessment [86]
	Fall history (8)	2 items
	Alcohol consumption (9)	3 items
	Stress (10)	4 items, Perceived Stress Scale (PSS) [87]
	Insomnia (11)	7 items, Insomnia Severity Index (ISI) [88]
	Menstruation cycle (12)	7 items
	General health and chronic disease, part 2 (19)	13 items
	Medication (20)	10 items
Anthropometry	BMI ^a^ (13)	Weight and height
	WHR (14)	Waist circumference/hip circumference
	Body composition (15)	Four-segment bioelectrical impedance analysis
Macrovascular-Health	Arterial stiffness (baPWV/CAVI) & blood pressure ^a^ (16)	Noninvasive vascular screening system
	Brachial endothelial function (17)	FMD by ultrasound
	Carotid-intima-media thickness (23)	2D ultrasound instrument
Cardiac Imaging	Systolic and diastolic structure and function (22)	2D echocardiography
Inflammation & Circulating CV Risk Factors	Cholesterol (TC, LDL, HDL), triglycerides (TGA), HbA1c, NT-pro BNP & etc. (21)	Venous blood samples
Physical Fitness Components	Gait (18)	Inertial sensor system
	Power of leg muscles (24)	Countermovement jump on a force plate
	Standing balance (25)	Tandem stance on a force plate
	Handgrip strength (26)	Handheld dynamometer
	Isometric leg strength (27)	Dynamometer
	Cardiorespiratory fitness (28)	Cardiopulmonary exercise testing with breath-by-breath gas analysis
Starting the day after visit 1 for 14 days		
Physical Activity	Objective physical activity	Wrist-worn triaxial accelerometer
Visit 2		
Microvascular-Health	Retinal arterial and venous diameters	Static retinal vessel analysis
	Retinal endothelial function	Dynamic retinal vessel analysis

^a^ Used for a check of inclusion criteria. Numbers in brackets indicate the precise sequence of data collection of visit 1

Abbreviations: *BMI* body mass index, *WHR* waist-to-hip ratio, *baPWV* brachial–ankle pulse wave velocity, *CAVI* cardio–ankle vascular index, *TC* total cholesterol, *LDL* low-density lipoprotein, *HDL* high-density lipoprotein, *FMD* flow-mediated dilation

### Physical fitness components

#### Endurance capacity: cardiopulmonary exercise testing

An exercise test until maximal exertion using an electromagnetically braked cycle ergometer (Ergoselect 200; Ergoline, Bitz, Germany) will be performed according to one of the following five protocols: a three-minute warm-up will be performed either unloaded with a load of 10 or 20 W for protocols 1 to 3 or with a load of 50 W for protocols 4 and 5; a warm-up will be followed by ramp protocol 1, 2, 3, 4, or 5 with a linear workload increase of 7, 10, 15, 20, or 30 W/min, respectively. The three-minute recovery phase will be performed at the same wattage as the warm-up. The protocol will be chosen to achieve a ramp duration of between six and 18 min [89, 90]. Pedalling cadence will be chosen by participants but is required to be more than 60 rpm.

Gas exchange and ventilatory variables will be analysed breath-by-breath continuously using a computer-based system (MetaMax 3B; Cortex Biophysik GmbH, Leipzig, Germany). Every test is preceded by a resting period of three minutes to reach steady-state conditions. The steady-state status will be analysed for the plausibility of V̇O_2_ (mL/min), V̇CO_2_ (mL/min), V̇E (L/min), ventilatory equivalents for oxygen and carbon dioxide (V̇E/ V̇O_2_ and V̇E/ V̇CO_2_, respectively), and end-tidal gas tensions for oxygen and carbon dioxide (mmHg). A trained and certified sports scientist continuously supervises the examination, and a physician is always available on request when testing CHF patients and participants older than 50 years. In the absence of chest pain and electrocardiogram (ECG) abnormalities, all tests will be continued until maximal exertion (i.e., volitional exertion, dyspnea, or fatigue). The capillary blood lactate concentration from the earlobe will be measured at rest, at maximum performance, and at one and three minutes after the end of the exercise test. The Borg scale [91] will be applied during warm-up and every two minutes thereafter until exhaustion. Before and during the test, patients will be encouraged to reach maximal exhaustion. All tests will be performed according to the current guidelines for exercise testing and in controlled humidity and temperature conditions [92]. Before each test, the equipment will be calibrated in standard fashion with reference gas and volume calibration. A standard 12-lead ECG will be obtained at rest, during the entire period of the exercise test, and for three minutes during recovery. The ECG will be equipped with an analysis of high-frequency components of QRS complexes (HFQRS) to improve the diagnostic value of exercise ECG [93].

V̇E (L/min), V̇O_2_ (mL/min), and V̇CO_2_ (mL/min) will be acquired on a breath-by-breath basis and averaged over 10-s intervals. V̇O_2_peak will be defined as the highest 30-s average of V̇O_2_ at any point of the test. The V̇E/maximal voluntary ventilation (MVV) will be calculated as peak V̇E in relation to MVV, and MVV will be calculated as forced expiratory volume in one second (FEV1) multiplied by 40 [92].

Blood pressure will be measured manually during warm up, every two minutes during the ramp protocol, immediately before maximal exertion, and during the recovery phase. Arterial oxygen saturation will be recorded continuously using a pulse oximeter (Masimo Corporation, Irvine, CA, USA).

Blood lactate concentration in mmol/L will be measured from 10 μl capillary blood drawn from the ear. The analysis of blood lactate concentrations will be done via the SuperGL Ambulance (Hitado Diagnostic Systems, Moehnesee, Germany) immediately after the last blood sample is drawn.

#### Muscle strength: isometric leg strength, countermovement jump, and grip strength

Isometric leg strength will be measured in both legs simultaneously, using an analogue dynamometer (TTM Muscular Meter, Tokyo, Japan). Participants will be instructed to lift the bar upward with maximum force [94] using only their legs and keeping their back straight. The test will be performed at a knee angle of 110°. This test examines isometric strength, predominantly of the quadriceps and hip extensors.

The countermovement jump will be performed on a force plate (Leonardo Mechanograph®, Novotec Medical, Pforzheim, Germany) to measure peak power. The instruction will be to jump with the head and chest as high as possible, thus producing the maximum elevation of the centre of mass. Participants who are unable to jump will be instructed to push as fast and hard as possible in order to generate power on the plate. This procedure can be performed by any participant without security concerns. Each participant will perform three trials. The most critical outcome parameter of this test is the maximum power output (peak power) normalized to the body weight of the participant. The method has been validated in young and older adults [95].

Hand grip strength will be measured using a handheld dynamometer (Leonardo Mechanograph GF; Novotec Medical GmBH, Pforzheim, Germany). Participants will perform the test standing, using the dominant hand. Three attempts will be performed with participants in a standing position with their elbow in full extension, resting for 60 s between attempts [96]. Grip span will be adapted to the individual hand size [97]. Maximal achieved grip strength (kg), and rate of force development (RFD) will be used for the analyses. RFD describes the capacity to produce voluntary activation in the early phase of contraction (first 75 ms) [98],

#### Neuromuscular coordination: balance test and gait analysis

A force plate (Leonardo Mechanograph®; Novotec Medical, Pforzheim, Germany) will be used to assess the centre of pressure during an upright static tandem stance. Patients will be asked to maintain an upright position with their knees slightly flexed (~ 10°), hands at their side, and their gaze straight ahead for 10 s on a cross 1.5 m away on the wall. The cumulative sway path during this period will be registered and serves as a measure of postural control. To minimize bias through potential learning effects, the test will be repeated three times. Additionally, failed attempts will be recorded should a patient seek support [99]. This test has been shown to have a good degree of reliability [100].

An inertial sensor system (Physilog®; GaitUp, Lausanne, Switzerland) will be used to analyse participants’ gait. The lightweight sensors, integrating a three-axis accelerometer and a gyroscope, will be attached to the participants’ feet. Participants will be asked to walk at their habitual speed on a 20-m walkway (two-way, one attempt). The sensor system has previously shown good validity and reliability in assessing speed, spatiotemporal parameters, and foot kinematics of walking [101, 102].

### Vascular aging

#### Macrovascular measurements: arterial stiffness measurement and flow-mediated dilation

Arterial stiffness will be measured as baPWV using a noninvasive vascular screening system (VaSera VS-1500 N; Fukuda Denshi, Tokyo, Japan). The participants are requested to fast for at least two hours before the examination and to abstain from alcohol and caffeine on the day of the examination. After 10 min of rest in a quiet, dark room with ambient temperature (23 °C–26 °C) in a supine position, measurements will be performed. Blood cuffs will be placed above the left ankle and the left upper arm. A foot-to-foot method will be used to determine the time delay of the pulse wave from the heart to the ankle. Using a height-based formula, vascular length between the heart valve and the ankle artery will be estimated as baPWV by the VSS-30 software (Fukuda Denshi, Tokyo, Japan) [103]. In addition, the central PWV will be calculated by the application of the ARCSolver algorithm to pulse wave signals acquired with the VaSera VS-1500 device to estimate central systolic blood pressure (cSBP) and aortic PWV [104]. Peripheral and central blood pressure, pulse wave reflection as augmentation index, and arterial stiffness are commonly used as independent predictors to assess CV risk [59].

The FMD examination follows the measurement of arterial stiffness after at least 15 to 20 min of rest in the supine position. An occlusion cuff will be wrapped around the right forearm with the proximal edge of the cuff at the elbow. Using a high-resolution ultrasound linear array transducer, longitudinal images of the right brachial artery (typically located at 3–15 cm above the elbow) are recorded at the baseline and after cuff deflation following suprasystolic compression (50 mmHg over the systolic blood pressure value) of the right forearm for five minutes, until three minutes after deflation. FMD is measured by the A-mode waves as a signal of the intima-media complex (Unex Corporation, Nagoya, Japan). Furthermore, blood flow velocity will be measured according to pulsed Doppler values at the baseline and during peak hyperaemic flow. Inter-reader and intersession reliability of the FMD measurement was shown to be acceptable [105, 106].

#### Microvascular retinal measurements: static retinal vessel analysis and dynamic retinal vessel analysis

Static retinal vessel diameters will be analysed at a second visit using the Dynamic Vessel Analyser (DVA; IMEDOS Systems, Jena, Germany) as previously described [107]. Twenty minutes after pupil dilatation, measurements of retinal arteriolar and venular diameters will be performed. Three valid images are taken from the retina of the right eye with an angle of 30° and with the optic disc in the centre, per visit. Retinal arterioles and venules, coursing through an area of 0.5 to 1 disc diameter from the optic disc margin, will be identified semiautomatically at higher magnification using special analysis software (Vesselmap 2; IMEDOS Systems, Jena, Germany). Diameters will be averaged to central retinal arteriolar and venular equivalents (CRAE and CRVE), using the Parr–Hubbard formula described elsewhere [108], and the AVR will be calculated from the CRAE and CRVE. The reliability of this method is high, with interobserver and intraobserver interclass correlation coefficients for arteriolar and venular diameter measurements ranging from 0.78 to 0.99 [108, 109].

Dynamic retinal vessel imaging will be performed in one eye with the same DVA device as previously described [107]. Arteriolar and venular vessel branches measuring approximately 1 mm in length, located in the upper temporal quadrant 1 to 2 optic disc diameters away from the optic disc edge, will be assessed. Retinal vessels will be stimulated with a flickering light relying on the principles of neurovascular coupling. The vascular stimulation and its underlying principles have been previously described [110]. In brief, an optoelectronic shutter is inserted into the retina camera in place of an additional optical filter. The shutter interrupts the observation light (530–600 nm) with a frequency of 12.5 Hz and provides a sequence of one normal illuminated and one dark single frame at a video frequency of 25 Hz. The measurement of the baseline vessel diameter for 50 s is followed by three cycles of 20 s of flicker provocation and 80 s of observation. The total duration of the measurements, including baseline and observations between flicker provocations, amount to 350 s.

### Echocardiography

Echocardiography will be performed with the Fukuda UF 760 ultrasound scanner (Fukuda Denshi, Tokio, Japan) and with an SA16 (2–5 MHz) transducer (Fukuda Denshi, Tokio, Japan) by experienced physicians according to the recommendations of the American Society of Echocardiography and the European Association of Cardiovascular Imaging [111]. In brief, echocardiography includes the determination of EF (two-dimensional, modified Simpson rule), PW-Doppler E- and A-waves at the mitral wave; Tissue Doppler E´-, A´-, and S´-waves at the septal and lateral sites of the left chamber; and left atrial diameter (m-mode, parasternal long-axis view, and apical four- and two-chamber views). For the measurement of left ventricular mass, chamber dimensions and wall thicknesses will be acquired from the parasternal long- and short-axis views using targeted m-mode echocardiography at the level of the mitral valve leaflet tips at end diastole, with the m-mode cursor positioned perpendicular to the septum and the left ventricular posterior wall. All measurements will be analysed with a computerized review station (EZ Desk; Fukuda Denshi, Tokyo, Japan) as done in a previous study [60], with our lab as the core reading centre.

### Intima-media thickness

Carotid intima-media thickness (CIMT) will be measured with the Fukuda UF 760 ultrasound scanner (Fukuda Denshi, Toyko, Japan) with a FUT-LA385-12P (8–13 MHz) transducer (Fukuda Denshi, Tokyo, Japan) according to standard procedures previously used in the SAPALDIA-cohort study [112]. Automatic measurements will be limited to the right common carotid artery to assess CIMT and carotid stiffness according to established procedures [113, 114].

### Inflammation and circulating cardiovascular risk factors

Blood samples are drawn by venipuncture of the cubital fossa of the right or left arm by trained medical staff in fasting status (at least three hours). The total volume of blood samples taken are 2 × 2.7 mL potassium-EDTA, 2 × 7.5 mL serum-monovette, and 1 × 7.5 mL Li Heparin. Blood samples are immediately centrifuged, and the plasma aliquots are frozen at a temperature of − 80 °C. Planned blood analysis for basic characterization of risk factor profile include total cholesterol, low- and high-density lipoprotein, triglycerides (colorimetric tests), and haemoglobin A1c. N-terminal pro-hormone B-type natriuretic peptide will be analysed by standard laboratory assays with the Cobas analyser (Cobas 8000; Roche Diagnostics, Basel, Switzerland).

### Anthropometry and questionnaires

All questionnaires will be digitally recorded. The standardized questionnaires used in this study are the following: the European Health Interview Survey-Physical Activity Questionnaire (EHIS-PAQ) [84], Global Physical Activity Questionnaire (GPAQ) [85], Physical Activity Readiness Questionnaire (PAR-Q), Munich Chronotype Questionnaire (MCTQ) [82], Health-related Quality of Life questionnaire, short form (SF-8) [83], Perceived Stress Scale (PSS) [87], Insomnia Severity Index (ISI) [88] and a shortened version of the University of Alabama at Birmingham (UAB) Study of Aging Life-Space Assessment [86]. General health and medical conditions, use of a walking aid, frequency of falls (12-month recall) [115], medication use, alcohol consumption, menstruation cycle, residential area, use of transportation, and socioeconomic status will also be assessed by self-report.

Body composition will be analysed by four-segment bioelectrical impedance analysis using the InBody 720 (Inbody Co. Ltd., Seoul, South Korea). Measurement of appendicular muscle mass with InBody 720 is acceptable as compared with dual-energy x-ray absorptiometry analysis [116, 117]. Participants will refrain from any intense physical activity for 24 h prior to measurement, will fast for a minimum of two hours, and will be asked to void their bladder before the measurement.

### Physical activity

Physical activity will be objectively measured over 14 days [118, 119] using a wrist-worn triaxial accelerometer (GeneActive Activinsights Ltd., Kimbolton, UK). GeneActive accelerometers have previously been validated [120]. The device will be attached to the participant’s nondominant wrist and samples data at a frequency of 50 Hz. Participants will be asked to wear the device continuously during day and night in their free-living conditions. Accelerometry data will be exported using the GENEActiv software version 2.9 (GENEActiv Activinsights Ltd., Kimbolton, UK) and will be collapsed into 60-s epoch files.

## Statistical analysis

Participant characteristics will be analysed descriptively. The distribution of continuous variables will be inspected graphically and characterized with either the mean and SD or with the median and interquartile range. Categorical variables will be presented as absolute and relative frequencies. Age trajectories of components of physical fitness (endurance capacity, muscular strength, and neuromuscular coordination) will be analysed over decades, using multiple linear regression models [121]. Model diagnostics will include residual diagnostics and assessment of multicollinearity [122]. If necessary, standard errors, confidence intervals, and *p*-values will be adjusted for heteroscedasticity [123]. Model selection will be done based on the Akaike Information Criterion (AIC) [124]. Models will be adjusted for potential confounders such as body mass index, blood pressure, and sex. Both linear and nonlinear trajectories will be considered [125]. Sensitivity analyses will include the inclusion of interaction terms with the decade term to detect potential differences between subgroups of trajectories of components of physical fitness. Multiple imputation using chained equations (MICE) will be used for variables with a high proportion of missing data [126]. Essential differences between multiple imputation analyses and complete case analyses will be discussed. All statistical tests will be two-sided with a significance level of 5%.

### Analyses with health distance

We will explore new avenues in computing the health distance (HD) for healthy individuals and heart failure patients based on a methodology designed initially for longitudinal studies applying it to cross-sectional data [127–129]. In their research, Arbeev et al. [128] describe the analogue procedure on the optimal versus realized trajectories of physiological dysregulation in aging organism and their relation to sex-specific mortality risk in the framework of a mathematical model of aging and mortality [130]. A recent research [131] further elaborated the approach applying it to data on onset of and survival from aging-related diseases and illustrated that such measures can potentially be used as a preclinical indicator of transition from healthy to unhealthy state. This approach measures physiological dysregulation based on deviations of multiple biomarker profiles from their ‘reference’ values. In the present study, this method (originating from [129]) will be applied to all components of physical fitness, to the vascular imaging biomarkers, and to the established traditional risk factors.

The HD is defined as the statistical (Mahalanobis) distance [132] constructed for the joint distribution of multiple biomarkers [129]: $HD\left({X}_i\right)=\sqrt{{\left({X}_i-\overline{X}\right)}^T{S}^{-1}\left({X}_i-\overline{X}\right)}$.

where *X*_*i*_ is a vector of biomarkers measured in the individual *i* and $\overline{X}$ and *S* are the vector of means and the variance–covariance matrix, respectively, in some ‘reference’ population from which the distance is computed.

We will use the younger part (defined using different cut-off ages, see discussion in the paragraph preceding section *Sample size calculation*) of the healthy (C-Health) population as the ‘reference’ population. For this group, we will compute the means and variance–covariance matrix for the sets of measured quantitative biomarkers (see measurements), separately for females and males. Next, the health distances for the C-Health population and for ‘cases’ (i.e., age-matched patients with heart failure) will be computed using the above equation, with respective sex-specific means and variance–covariance matrices $\overline{X}$ and *S* taken from the ‘reference’ population, and the null hypothesis that the distances in these two groups are equal will be tested.

A baseline scenario will allow us to use all available quantitative biomarkers to define the health distance. We will also specify different ‘domain-specific’ subsets of biomarkers (e.g., those corresponding to endurance capacity, muscular strength, neuromuscular coordination, etc.) as well as separate biomarkers to identify which (sets of) biomarkers produce larger distances from the ‘reference population.’ As the approach assumes multivariate normality of the biomarkers, we will transform each biomarker, as necessary, using the appropriate transformation (e.g., the Box–Cox transformation) and then standardize them to a zero mean and unit variance to ensure that all transformed biomarkers are on the same scale in the analyses. The approach also assumes there are no missing data; however, in case of missing data for some biomarkers, we will perform the analyses imputing missing values of biomarkers using a fully conditional specification method of multiple imputation [133], and apply the standard Rubin’s rules for statistical inference.

We also note that many biomarkers (e.g., blood pressure, cholesterol, etc.) change nonlinearly with age, as shown in prior publications [134–137] that found a ‘bell-shaped’ relationship of the biomarkers with age (i.e., increasing and then declining). As a result, some biomarkers at the oldest ages (90–100 years) may be closer to the ‘reference’ values in the middle ages versus the values in the old ages (e.g., 70 years). Therefore, the relationship between the dynamics of biomarkers and aging is very complex and nonlinear (see, e.g., extensive discussion on the topic in a recent review paper [127]). The COmPLETE study, where biomarkers will be measured for individuals in broad age ranges spanning decades, provides extensive opportunities to check the possible nonlinearity of the patterns of the health distance with age. Although this is a cross-sectional study, it is an important contribution to this area of research, as this topic is still largely underexplored [127]. In this project, we will compute the health distances using different age groups to define the ‘reference’ populations and compare the health distances computed for different ‘reference’ groups (or for different decades of age for the same ‘reference’ group) for different combinations of biomarkers to tackle the question of the complexity of age-related changes in biomarkers and health.

### Sample size calculation

We used simulations to estimate the required sample size for a linear regression model assessing the trajectory of V̇O_2_peak in seven 10-year decades [138]. Based on prior studies in healthy and active individuals, the standard deviation of the V̇O_2_peak was assumed to be 6.0 mL/kg/min for each decade [139–141]. Assuming a mean decrease of 5.0 mL/kg/min per decade [139–141], 34 participants per sex were determined to be needed to achieve the required power of 80% (*p* = 0.05). Additionally, 17 men and 17 women per decade were deemed required to detect a difference between men and women regarding the trajectories of V̇O_2_peak.

We also conducted power analyses for the studies with the health distance assuming that the distribution (standard deviation = 1.077) and the dynamics (the annual rate of change = 0.034) of this measure resemble those in the Framingham data [128]. With the proposed sample size of 80 heart failure patients and 210 (70 participants per the decades 50–59, 60–69, and 70–79) age-matched healthy participants, the study will have the power of 80% to detect the difference between the health distances in these two groups of about 0.4 units for the two-sided test (about 0.35 units for the one-sided test). The Framingham data confirm that such a difference would correspond to differences between health distances for individuals who are about 12 (SD: 10) years of age apart.

## Potential pitfalls and limitations

### Pitfalls

The recruitment of 70 healthy individuals in the category of 80 years and older for C-Health and 80 participants with heart failure for C-Heart constitutes a challenge.

### Limitations

We are aware that our study has some limitations. It is clear that the data acquired from C-Health will not be fully representative of the Swiss population, since we will only examine a circumscribed population sample from the Basel region. Selection bias through our recruitment strategy, considering an inclusion rate of 3 to 5% of invitations sent, cannot be excluded and it is likely that our study participants will have an improved physical function and a better health status versus individuals who received an invitation but did not take part in the study. However, since we are not trying to recruit a representative sample of Swiss or Basel citizens but rather a sample of healthy male and female across the age groups, we think potential selection bias is a negligible problem for our aims.

Selection bias, however, might become more evident with ascending age decades in C-Health. A negative correlation between physical activity and physical fitness and some exclusion criteria such as high blood pressure, a body mass index of ≥30 kg/m^2^, or chronic disease can be assumed. The individuals meeting the inclusion criteria might, therefore, be more fit and active than their age groups members not meeting the inclusion criteria. This effect could be more significant in the older age groups and decrease the effect of age on the measured physical fitness parameters. However, a goal of our study is to describe the reduction in fitness markers only due to aging and not a chronic disease; this potential limitation could also be seen as a strength.

Further limitations are circadian, seasonal, and hemodynamic fluctuations, which influence vascular as well as physical fitness markers. The exact time of day and the season will be recorded to control for these fluctuations.

Further, there are some limitations concerning our measurement methods. We do not use the gold standard measurement for the assessment of isometric leg strength, which would either be by an isokinetic dynamometer or a custom-built isometric strength testing chair with an external analogue-to-digital converter. Further, we decided to use only one balance task. The tandem stance could be too easy for young individuals and cumulative change in sway paths may not discriminate between good and poor neuromuscular balance. On the other hand, this task could already overburden older participants who might be unable to perform the tandem stance for 10 s without seeking support.

We are aware that this is a cross-sectional observational study, and reverse causation is possible. However, no previous data exist regarding different comprehensive components of physical fitness, and potential follow-up in five years may allow for strengthening of the findings.

## Discussion

The COmPLETE study with its design will allow for the investigation and characterisation of the physical fitness components of endurance capacity, muscle strength, and neuromuscular coordination in individuals without chronic diseases from the 20th to the 100th year of life as well as in patients with heart failure. Therefore, this study will construct a novel dataset with normal values for all major physical fitness markers in healthy individuals.

The additional comprehensive assessment of vascular biomarkers in these individuals offers the opportunity to discriminate within apparently healthy individuals. Separately, it allows for the investigation of the mechanisms of aging and the role of physical fitness components and physical activity on vascular markers in various vasculature beds in health and heart failure.

Furthermore, the COmPLETE study may elucidate new approaches in diagnosis with its combined and extensive assessment of physical fitness and vascular biomarkers. This will enable us to find the most suitable diagnostic markers for CV risk and heart failure.

According to the inverse association of several vascular biomarkers with physical fitness components (endurance capacity, muscle strength, and neuromuscular coordination), individuals with excellent vascular health markers may have even better physical fitness than those with attenuated vascular health within the C-Health sample. The age-matched comparison with patients with different stages of heart failure may provide an estimate of the health distance of different fitness parameters to healthy individuals.

Health distance provides a new complex measure of aging-related decline in the adaptive capacity of the organism by comparing the values of the physiological or biological “norms” (C-Health) with those with prevalent heart failure (our example) [128].

The COmPLETE study shall provide a better understanding of which functional characteristics should be specifically targeted in primary and secondary prevention to achieve an optimal healthspan.

## Data Availability

not applicable.

## Abbreviations

AIC: Akaike information criterion
AVR: Arteriolar-to-venular diameter ratio
baPWV: Brachial–ankle pulse wave velocity
BNP: B-type natriuretic peptide
C-Health: COmPLETE-Health
C-Heart: COmPLETE-Heart
CHF: Chronic heart failure
CIMT: Carotid intima-media thickness
CPET: Cardiopulmonary exercise testing
CRAE: Central retinal arteriolar equivalents
CRVE: Central retinal venular equivalents
cSBP: Central systolic blood pressure
CV: Cardiovascular
DVA: Dynamic vessel analyser
EF: Ejection fraction
EOV: Exercise oscillatory ventilation
FEV1: Forced expiratory volume in one second
FMD: Flow-mediated dilation
HD: Health distance
HFmEF: Heart failure with mid-range ejection fraction
HFpEF: Heart failure with preserved ejection fraction
HFQRS: High-frequency components of QRS complexes
HFrEF: Heart failure with reduced ejection fraction
LVEF: Left ventricular ejection fraction
MICE: Multiple imputation using chained equations
MVV: Maximal voluntary ventilation
NYHA: New York heart association
OUES: Oxygen uptake efficiency slope
PETCO2: Partial pressure of end-tidal CO2
PWV: Pulse wave velocity
RFD: Rate of force development
SCORE: Systematic coronary risk evaluation
SD: Standard deviation
V̇E/V̇CO_2_ slope: Relationship of the ventilation to carbon dioxide production slope
V̇O_2_peak: Peak oxygen uptake
